# Endoscopic Transsphenoidal Surgery for Cushing’s Disease: A Review

**DOI:** 10.7759/cureus.5254

**Published:** 2019-07-27

**Authors:** Mirza Zain Baig, Altaf Ali Laghari, Aneela Darbar, Umm E Hani Abdullah, Sumiya Abbasi

**Affiliations:** 1 Surgical Oncology, Rudy L. Ruggles Biomedical Research Institute, Danbury, USA; 2 Neurosurgery, Aga Khan University Hosiptal, Karachi, PAK; 3 Neurosurgery, Aga Khan University Hospital, Karachi, PAK; 4 Epidemiology, Liaquat College of Medicine & Dentistry, Karachi, PAK

**Keywords:** cushing disease, endoscopic endonasal, trans-sphenoidal surgery, operating microscope

## Abstract

Ever since the 1960s, transsphenoidal surgery has been the modality of choice for treating Cushing’s disease. Subsequent visualization of the pituitary fossa and sphenoid sinus may be done either with the operating microscope or with the relatively new endoscope. The endoscope due to its panoramic view allows greater visualization as compared to the operating microscope. It confers greater access to the cavernous sinus, sella, suprasellar, and parasellar regions and accommodates higher magnifications. It is bi-dimensional, however as opposed to the operating microscope that provides a three-dimensional view and allows greater depth perception. This article provides a comprehensive review of the advantages and disadvantages of the endoscope and compares it to the operating microscope. We hope this article will prove useful to both clinicians and academicians alike in their approach and management of Cushing’s disease.

## Introduction and background

Cushing syndrome is defined as a state of prolonged hypercortisolism and its accompanying manifestations. When attributable to a pituitary origin, typically a pituitary adenoma or rarely a carcinoma, it is known as Cushing’s disease - named after and first described by Dr. Harvey Cushing in 1932 [1-4].

Cushing’s disease is the most common cause of endogenous hypercortisolism [4-8]. It is a rare disorder that has an incidence of 1.2 to 2.4 cases/million/ year [9-10]. Its estimated prevalence is nearly 40 cases per million [9]. It is associated with considerable morbidity and mortality [3]. Five-year mortality rates are estimated to be 50% [3,10]. Its presenting features and long-term complications include rapidly increasing weight, truncal obesity, abdominal striations, moon facies, buffalo hump, proximal myopathy, hypertension, easy bruising, depression, reduced immunity and metabolic disturbances such as metabolic syndrome, diabetes mellitus, deranged lipid profiles, and osteoporosis [3-4,6,8,10-13].

The first report on the transsphenoidal route to access the pituitary dates back to 1907 by Hermann Schloffer, after which further attempts at exploration were abandoned for nearly half a century [14-15]. It was during the 1960s that the transsphenoidal approach gained popularity and became the established treatment of choice for patients with Cushing’s disease [7-8,15-26]. This was due to the introduction of the operating microscope by Jules Hardy [14]. High remission rates coupled with few complications have encouraged the widespread use of the transsphenoidal route [8,16,20]. Only exceptionally, a transcranial approach may be needed [27]. In the 1990s, Jho pioneered an endoscopic transnasal technique that has become an alternative to the microscopic technique [14].

This article aims to compare the two techniques by summarizing the findings of recent clinical series published in literature with a special focus on the advantages of the endoscope along with any shortcomings when compared to the operating microscope. We hope this article will prove useful to clinicians and academicians alike in their approach and management of Cushing’s disease.

## Review

Traditionally the transseptal/translabial approach with the use of the operating microscope is the gold standard transsphenoidal approach [8,15,17,20,24]. It is associated with minimal morbidity and mortality [15]. However, with recent advances, the endoscope has come forward as an effective tool - one with the potential to perhaps surpassing the use of the microscope to become the modality of choice in Cushing’s disease [15,20-21,28-32]. Many studies throughout the literature comment on the endoscope’s ability to achieve better resection rates, lesser invasiveness, and fewer complications [7,17,20-22]. Advocates of the microscope, however, criticize its panoramic view for its lack of three-dimensional vision and depth perception, and the inability to conduct meticulous microsurgical procedures that comes with it [7,19,21]. Others, meanwhile favor the panoramic view as it leads to better visualization of the bony structures covering the carotid arteries and the optic nerve [6-7, 17-22,24,26,29-30,33-36].

Remission rates, reported in the literature for transsphenoidal surgery for Cushing’s disease vary between 42% and 95% [21]. The majority of remission rates lie between 70% and 85% with no significant improvement in the past years [21]. According to Qiao et al, there is no difference in remission rates between the endoscope and the microscope for Cushing’s disease [30]. There may be fewer recurrences with the endoscope but this advantage disappears when follow up time is taken into account [30].

Being a relatively newer innovation, there are only a few reports that look at the efficiency and prognostics of a purely endoscopic technique for Cushing’s disease. The effectiveness of pituitary surgery is evaluated by normalization of hormone levels and degree of tumor removal [15]. At the moment, data suggests that the endoscope is at least equivalent or in some cases even superior to the operating microscope [15,22]. Please refer to Table 1 for the salient features including remission rates, recurrences, complications, etc. of clinical series published in the literature on the use of the endoscope for Cushing’s disease [2,7-9,17-18,21,26,29,32,34].

**Table 1:** Summary of case series published on endoscopic transsphenoidal resection of Cushing’s disease that were available on PUBMED as full-text articles Abbreviations: MRI: magnetic resonance imaging, CSF: cerebrospinal fluid, DI: diabetes insipidus, ADH: antidiuretic hormone, GH: growth hormone

Paper	Patient characteristics	Modality	Findings	Complications
Natea -Maier et al, 2006 [7]	35 patients (25 females and 10 males). Mean age of 41.0 ±14.8	Endoscope	The remission rate of 77% after the first surgery and 83% after re-operation. The recurrence rate was 22.8%.	48% of the patients developed hypopituitarism. Severe epistaxis in one patient. 3 patients developed CSF leakage. 3 patients had polyuria, and 1 developed hyponatremia. 1 developed mild hyponatremia.
Dehdashti et al, 2007 [21]	25 patients (19 females and 6 males). Mean age of 42 ±2.5	Endoscope	The remission rate was 83%. None of the patients presented with recurrence at a median follow up of 17 months.	1 patient had a postoperative CSF leak. 1 patient developed transitory DI.
Starke et al, 2012 [34]	61 patients (52 females and 9 males). Mean age of 49 (14-63)	Endoscope	The immediate remission rate of 95%. The remission rate of 84% in patients with at least one year follow up. With additional adjuvant therapy, 94% successfully achieved remission. No significant difference in remission rates between microadenomas (93%), macroadenomas (77%) and MRI-negative Cushing’s (100%).	1 patient had a postoperative CSF leak. 1 patient presented with severe epistaxis.
Smith et al, 2012 [32]	72 patients- male to female ratio was 1:3.7. The median age of 40 years (31-50).	Operating microscope- 58 patients Endoscope- 14 patients	The initial remission rate was 72 %. The recurrence rate was 11%. The median time of recurrence after initial remission was 2.1 years. No significant difference between the operating microscope and endoscope.	3 patients developed meningitis. 1 patient developed sinusitis postoperatively. 1 patient had a septal perforation. 1 patient had a blocked lacrimal duct. Common complications seen were transient DI and postoperative CSF leak.
Wagenmakers et al, 2013 [29]	86 patients (72 women and 14 men). Mean age 42.3 ±14.9	Endoscope	Remission rate 60% in MRI-negative Cushing’s disease, 83% in microadenomas, 94% in noninvasive macroadenomas, and 40% in macroadenomas invading the cavernous sinus. The recurrence rate was 16% after 71+39 months of follow-up.	Postoperative bleeding from the sphenopalatine artery in 1 patient. Pulmonary embolism in 1 patient of persistent Cushing’s disease after surgery. Postoperative CSF leak in 4 patients. Transient DI in 4 patients. Transient hyponatremia due to inappropriate ADH secretion or relative glucocorticoid deficiency in 10 patients. Infection in 3 patients.
Berker et al, 2013 [8]	90 patients (79 women and 11 men). Mean age 38.74 ± 13.01	Endoscope	Remission achieved in 90 % of patients (86.9% microadenomas, 96.6% macroadenomas, 95.7% primary patients, 71.4% recurrent/ persistent disease. The recurrence rate was 5.6%. Reentered remission after reoperation	Intraoperative CSF leak in 8 patients. Out of which, 2 had a postoperative leak as well. Temporary DI in 7 patients Permanent DI in 1 patient. Postoperative meningitis after two weeks in 1 patient.
Storr et al 2014 [17]	Six pediatric patients (5 males and 1 female). Mean age 14.6	Endoscope	Remission achieved in 83.3% of the patients. No recurrence at mean 4.7 years follow up	Intraoperative sinus bleeds in 1 patient. Postoperative CSF leak in 1 patient. Panhypopituitarism in 1 patient. GH and gonadotropin deficiency in 1 patient.
Kuo et al, 2015 [26]	40 patients (38 females and 2 males). Mean age 41 ± 13		Remission achieved in 72.5% (81.8% microadenomas, 77.8% noninvasive macroadenomas, 44.4% macroadenomas that invaded the cavernous sinus. Recurrent/ persistent disease in 11 patients	CSF leak in 5 patients.
Sarkar et al, 2016 [18]	64 patients Mean age 31.9 ± 9.6	Endoscope	Remission in 79.7 % of the 59 cases followed up for >3 months and was superior for microadenomas (86.4 %) versus macroadenomas (55.6 %) and equivocal MRI adenomas (66.7 %).	Postoperative CSF rhinorrhea occurred in 5 patients. New endocrine deficits in 17.1 % of patients.
Cebula et al, 2017 [2]	230 patients. Mean age of 42 ± 13.5 years	Endoscope	Remission in 79.1% of patients after a median follow up of 21 ± 19.2 months. The remission rate was significantly increased for microadenomas and positive histology. The recurrence rate of 9.8% with a mean time 32.7±15.2 months.	Post-operative complication occurred in 77 patients (35.5%). Predominant postoperative complications were transient DI and intraoperative CSF leakage (22% and 12.6% respectively). The rate of long-term DI was 6.4%. Two cases of transient visual complications occurred. Four people had epistaxis.
Donofrio et al, 2017 [9]	709 patients (142 Cushing’s disease patients and 299 nonfunctioning pituitary adenomas).	Operating microscope	A remission rate of 80.3%.	Major complications reported in 7 Cushing’s disease patients (4.9%). Minor complications reported in 3 Cushing’s disease patients (2.1%). Postoperative DI reported was 10.6% and isolated hyponatremia reported was 10.6%.

Operative technique

**Figure 1:**
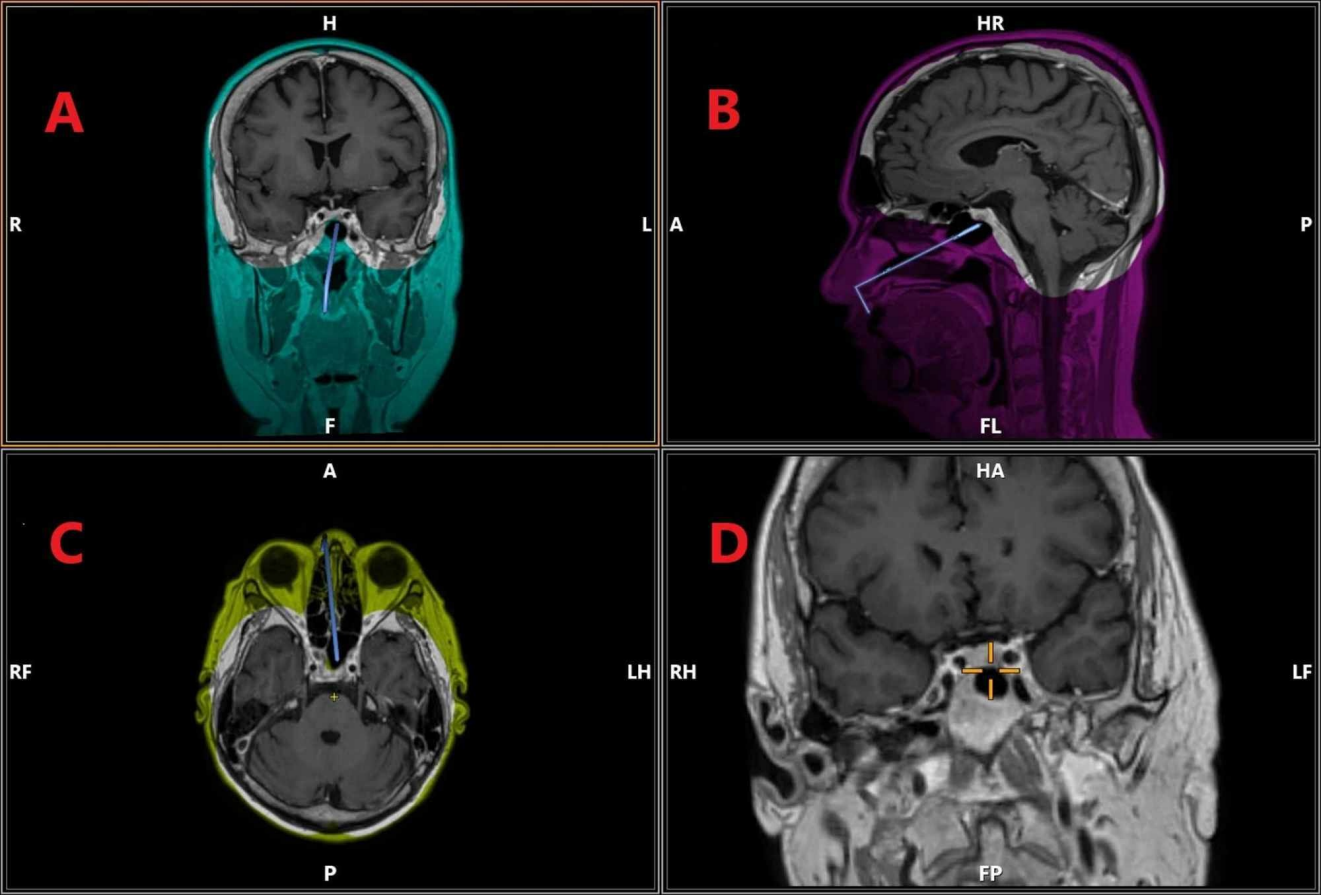
Neuronavigational planning of endoscopic transsphenoidal surgery in a patient with Cushing's disease. (A) T1 with contrast, coronal section. (B) T1 with contrast, saggital section. (C) T1 with contrast, axial section. (D) Magnified coronal section showing a tumor in sellar region, more on the right side The different color schemes in the figure hold no significance and are a result of the software used for neuronavigational planning

The procedure is done under general anesthesia. It is conducted in collaboration with an otolaryngologist. The patient is kept supine with the head maintained in a fixed position using a three-pin Mayfield clamp. The head of the bed is elevated. Frameless stereotaxy is used for neuronavigation (Figure 1).

Using the binostril, bimanual technique, the endoscope is inserted and the Hadad flap is raised. The sphenoid ostium is identified. The posterior septum is removed to expose the vomer. The sphenoid sinus is identified and the intersinus septum is removed. The anterior part of the sella is then opened using a drill and Kerrison rongeur.

After identifying the bony landmarks of the optic nerve, carotid artery, and opticocarotid recess, a disc dissector is used to remove dura from the bone of sella floor. The dura is opened and separated from the gland underneath using a micro dissector. Care should be taken not to coagulate dura as this may lead to white discoloration that may hinder tumor identification [19]. Once the bone has been removed, neuronavigation is used to locate the tumor. Resection is then carried out using a micro dissector, suction device, and ring curettes of varying diameters and orientations. The tumor is identified as a discolored gray region upon the orange-pink coloration of the gland.

The sellar defect is repaired using the Hadad flap followed by fibrin adhesive, Surgicell, and Gellfoam.

Video 1 reviews the operative technique for endoscopic transsphenoidal surgery.

**Video 1:** Endoscopic transsphenoidal removal of Cushing’s disease. Procedure done by authors AAL and AD. Commentary by author MZB. Abbreviations: AAL: Altaf Ali Laghari, AD: Aneela Darbar, MZB: Mirza Zain Baig

**Figure 2:**
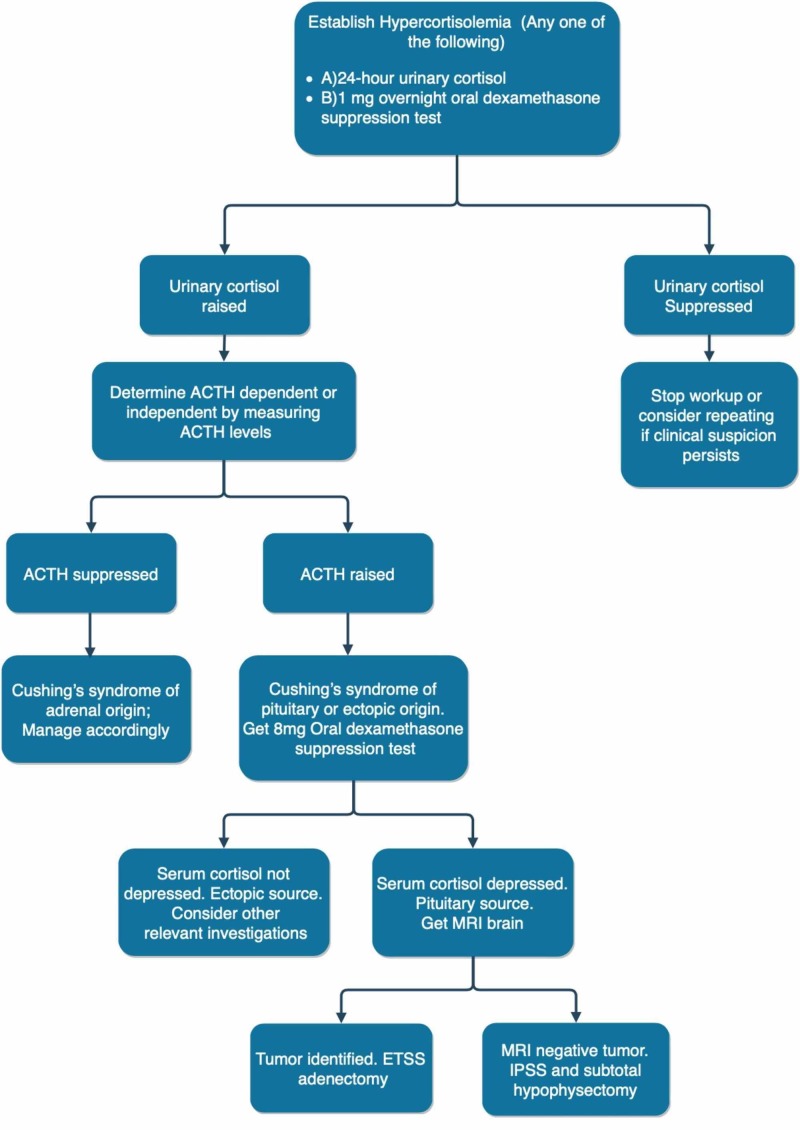
Diagnostic workup of Cushing’s syndrome Abbreviations: ACTH: adrenocorticotropic hormone; MRI: magnetic resonance imaging; ETSS: endoscopic transsphenoidal surgery; IPSS: inferior petrosal sinus sampling

In cases of negative magnetic resonance imaging (MRI), inferior petrosal sinus sampling (IPSS) is done to determine the lateralization of the disease. A selective adenectomy is done in cases where the tumor is identified on surgical exploration. Otherwise, subtotal hypophysectomy is done on the side of IPSS lateralization. Refer to Figure 2 for a summary of the workup done for Cushing’s syndrome.

Advantages of the endoscope

The hallmark feature of endoscopic transsphenoidal surgery is the superior view one has of the sphenoid sinus and the pituitary fossa [7-8,13,15,17,20,22,30,34,36]. It gives greater lighting hence contributing to the better visualization and - depending on the scope used - an ability to operate at an angle [7-8,13,15,21,30]. Its panoramic vision allows greater exploration of the sella, suprasellar, and parasellar regions including the cavernous sinus area - as opposed to the traditional microscope that allows visualization only in a straight line between the scope and the pathology being observed [7-8,13,15,20-22,30,34]. This means that the surgeon can now visualize tumors superiorly at the base of the third ventricle, inferiorly to the lower clivus, and laterally to the carotids and the cavernous [15]. The endoscope also allows higher magnifications, which make it an excellent choice for patients with Cushing’s disease which are typically small tumors.

Another significant advantage of the endoscope is that it allows the surgeon greater access to the cavernous sinus [30,35]. Previously tumor invasion of the cavernous sinus was considered a negative prognosticator and an absolute contradiction to surgery [35]. It has been rightfully called the anatomic jewel box by Parkinson due to the density of neurovascular structures within its dural walls [25]. With the development of the endoscope, however, this is no longer the case and tumors reaching into the cavernous sinus can be successfully operated on and removed using a 30-degree scope [35].

Since the endoscopic approach does not utilize transseptal dissection, there is less post-operative pain and discomfort [7-8,17]. Hospital stays are shorter with fewer complications [7-8,17,22]. In particular, there are decreased incidences of septal perforation, epistaxis, and transient Diabetes Insipidus (DI) with the endoscopic technique [8,13,22]. This leads to greater patient satisfaction scores. It is because of these reasons and the fact that it causes minimal skull base trauma that Storr et al [17] emphasize the use of endonasal endoscopic transsphenoidal surgery in pediatric age groups. In their case series, Storr et al. also report fewer PICU admissions and blood transfusions. Also, being minimally invasive, reoperation, when needed, is much simpler as compared to the microscope which unfortunately brings about a greater distortion of normal anatomy [8,22]. Additionally, the wider field of vision of the endoscope serves helpful during reoperation when normal anatomical landmarks have been disrupted [17,19,22].

Disadvantages of the endoscope

The operating microscope resorts to its three-dimensional vision and depth perception, giving the surgeon the ability to operate in three-dimensional space - a feature that is, unfortunately, missing in the endoscope [7-8,20,30]. The endoscope is bidimensional and hence does now provide any depth perception [7,20-21,30]. This is by far the biggest disadvantage of the endoscope as the lack of stereoscopic vision makes it difficult to discriminate adenomas from surrounding hypophyseal tissue [13]. Although 3D endoscopes have been developed and are available in the market to address this issue, their widespread adaptation and the subsequent results remain to be seen in future literature [30]. There is also the difficulty of manipulating tools through a narrow corridor [8,21]. However, both these issues can be overcome with surgeon experience [8,21]. Using a binostril, bimanual technique may also address this [8,21]. The learning curve involved in endoscopic transsphenoidal surgery has been investigated in the series by Chao-Hung et al. where the authors stratified their patients temporally and reported greater recurrences in earlier cases [26]. This corroborates the presence of a learning curve in endoscopic transsphenoidal surgeries that required experience and training to be acquired [13,26].

Concerns have also been raised regarding its lack of maneuverability as surgeons can only manipulate tools with one hand unless a holder is used [7]. The use of an assistant may, however, crowd the operative field [7].

Another disadvantage that has been reported is the increased incidence of extracranial manifestations [20]. These include nasal crusting and synechiae formation [20]. This may be a direct result of the repetitive passage of instruments in the nasal cavity [20]. Postoperative nasal debridement is usually required [20]. 

There is also increased incidences of vascular complications and post-operative cerebrospinal fluid (CSF) leaks with the endoscope [13]. The increase in vascular complications may be attributed to the fact that the surgeon may attempt more radical tumor excision with the endoscope by virtue of the increased view.

Systemic review and meta-analysis

In our review of the literature, we found several systemic reviews and metanalysis that compared microscopic and endoscopic surgical techniques and prognostics in a heterogeneous population of patients with various pituitary adenomas [15,20,22,28]. We found two studies that were accessible as full-text articles that compared the two surgical techniques in the setting of Cushing’s disease only [13,30]. The findings of all these studies are summarized in Table 2 [13,15,20,22,28,30].

**Table 2:** Summary of systemic reviews and metanalysis published on endoscopic versus microscopic transsphenoidal resection of Pituitary adenomas that were available on PUBMED as full-text articles Abbreviations: DI: Diabetes Insipidus, CSF: cerebrospinal fluid, SIADH: syndrome of inappropriate antidiuretic hormone

Paper	Number of studies and patients assessed	Findings
Rotenburg et al, 2010 [28].	11 studies	Fewer complication rates in endoscopic surgeries. Differences in septal perforations were found to be insignificant in three studies. Decreased operating times, lumbar drains, immediate postoperative DI, rhinologic complications, length of hospital stay, and pain in endoscopic approach. Degree of tumor resection and change in post-operative hormone levels comparable in both techniques.
Goudakos et al, 2011 [15].	11 studies 806 patients (369 had endoscopic surgery and 437 had microscopic surgery)	66% remission rate in the endoscopic group versus 60% in the microscopic group. Degree of tumor resection comparable in both techniques. No significant difference between the rates of CSF leaks between the endoscope (19.5%) and the microscope (14.4%). Significantly shorter hospital stays with the endoscopic technique (3.7 – 4.4 days) versus microscopic technique (5.4 – 5.7 days).
Ammirati et al, 2012 [20].	24 cohort studies 1670 patients (702 had endoscopic surgery and 968 had microscopic surgery)	Higher rates of vascular complications with the endoscope
Esquenazi et al, 2017 [22].	21 studies 940 patients (292 had endoscopic surgery and 648 had microscopic surgery)	Transient DI higher in the endoscopic group (6.3%) versus the microscope (5.0%). No cases of permanent DI in the endoscopic group, while 2.8% of patients in the microscopic group had permanent DI. Higher rates of postoperative pituitary insufficiency in the endoscopic group (7.9%) versus microscopic group (5.2%) Higher rates of cranial nerve palsy in endoscopic surgery (1.4%) than in microscopic surgery (0.8%). Higher rates of CSF leaks in the endoscopic group (4.4%) versus in the microscopic group (2.1%). No mortality in the endoscopic group whereas 7 people died in microscopic with a pooled proportion of 1.5%.
Broerson et al, 2018 [13].	97 studies 6695 patients (984 had endoscopic surgery and 5711 had microscopic surgery)	Similar remission rates (80%) for both techniques. Hydrocortisone dependency was seen in 39.3% patients in microscopic surgery and 33.5% after endoscopic surgery. Similar recurrence rates (10%) for both techniques. Fewer rates of CSF leaks with microscopic surgery (4.0%) than in endoscopic surgery (12.9%). SIADH, bleeding and permanent DI were seen slightly less often in patients after microscopic surgery, than in patients after endoscopic surgery. Transient DI was reported more often in patients after microscopic surgery (21.7%) than in endoscopic surgery (11.3%). Recurrence rates 17.0 % after microsurgery and 1.5% after endoscopic surgery.
N Qiao, 2018 [30].	24 studies 1670 patients (702 patients had endoscopic surgery and 968 microscopic surgery)	No significant difference in remission rates between endoscopic surgery (79.7%) and microscopic surgery (76.9%). Recurrence rates for endoscopic surgery were 11.0% and for microsurgery, 15.9% Proportion of remission in micro-adenomas was statistically significantly higher in the endoscopic group (87.3%) than in the microscopic group (79.3%).

## Conclusions

According to the literature published to date, an endoscope is an effective tool in transsphenoidal surgeries. Its superior view along with better patient prognostics establish it as a superior modality for Cushing’s disease when compared with the microscope. Significant limitations need to be considered, however, as there exists a learning curve for surgeons using the endoscope. Lack of maneuverability and extra-cranial complications need to be addressed as well. In the future, 3D endoscopes may perhaps become a mainstream modality. The operative microscope can till then be utilized upon the surgeon’s discretion.
